# Determinants of health-related quality of life in children and adults with autoinflammatory diseases

**DOI:** 10.1186/1546-0096-13-S1-P174

**Published:** 2015-09-28

**Authors:** G Erbis, T Sergiichuk, SM Benseler, S Hansmann, J Kuemmerle-Deschner

**Affiliations:** 1 University Hospital Tuebingen, Department of Pediatrics, Division of Pediatric Rheumatology, Tuebingen, Germany; 2 University of Calgary, Rheumatology, Alberta Children's Hospital, Calgary, Canada

## Background

Familial Mediterranean fever (FMF) and cryopyrin-associated periodic syndrome (CAPS) are rare inherited autoinflammatory diseases (AID). Chronic inflammation may result in severe organ damage. Beyond the physical burden of the disease, its impact on psychosocial well-being affects all areas of life. Patients may encounter rejection resulting in isolation and the risk of psychological disorders. While the positive effects of IL-1 inhibition on the physical aspects of AID are well documented little is known about the psychosocial condition of these patients. Therefore, the aim of the study was to evaluate HRQL in patients with FMF and CAPS.

## Patients and methods

A single centre study of consecutive patients diagnosed with autoinflammatory diseases age ≥ 4 years was performed. Semi-structured interviews focussing on domains of burden of disease, activities of daily life, family, school and job, participation in social life and self-management were conducted. In addition, patients completed validated HRQL questionnaires including KINDL-R (children) and SF-36 (adults). Questionnaires were analyzed using descriptive statistics. Results were correlated with patient-related variables.

## Results

Interviews were conducted with 55 patients, 24 males and 31 females. Age distribution: 10 children age 4-7 years, 30 adolescents age 8-18 years and 15 adults. Diagnoses: FMF in 21, CAPS in 30 and unclassified AID in four. A total of 80 questionnaires were completed by affected children (7, 9%), adolescents (24, 30%), and adults (21, 26%) in addition to 28 unaffected parents (35%).

Overall, the patients' social well-being was impaired. The experience of not being believed was rated worst by almost all patients. Lack of understanding by doctors on their odyssey to diagnosis was experienced by 70%. Challenges in school and job were: above average times of absence (67%), impaired ability to concentrate (80%) and limited productivity (65%). The discrepancy between self-perception (feeling ill very often) and perception of others (not noticing the patients' disease) causes self-doubt (30%).

## Conclusion

Children and adults with autoinflammatory diseases report significantly impaired HRQL. Patients identified challenges in school and job as the key concern. Targeted interventions like school visits by trained social workers may address this area of need.

